# Severe respiratory distress secondary to pharyngeal perforation during endoscopic gastrostomy tube removal: a clinical case report

**DOI:** 10.3389/fgstr.2023.1191199

**Published:** 2023-06-05

**Authors:** Dima Siblani, Laure Stiel, Stéphanie Husson-Wetzel, Pierre Barsotti

**Affiliations:** ^1^ Service de Reanimation Medicale, Groupe Hospitalier de la Région de Mulhouse et Sud Alsace, Hôpital Emile Muller, Mulhouse, France; ^2^ Service de Gastroenterologie, Groupe Hospitalier de la Région de Mulhouse et Sud Alsace, Hôpital Emile Muller, Mulhouse, France; ^3^ Service de Chirurgie Générale Digestive et Endocrine, Groupe Hospitalier de la Région de Mulhouse et Sud Alsace, Hôpital Emile Muller, Mulhouse, France

**Keywords:** percutaneous endoscopic gastrostomy (PEG), enteral nutrition, ablation technique, respiratory insufficiency, pneumothorax, pneumomediastinum

## Abstract

We present the case of a 60-year-old patient with advanced chronic obstructive pulmonary disease (COPD), who presented for planned endoscopic removal of her gastrostomy feeding tube, which was inserted for nutritional status optimization prior to lung transplantation. The procedure was complicated by accidental blockage of the device at the pharyngeal level, causing a transmural laceration. Rapid respiratory distress developed with subcutaneous emphysema that led to the intubation of the patient. A new endoscopic retrieval was attempted but failed, and the patient was sent to the operating room after a cervical and thoracic CT scan that showed the blocked piece in the cervical wall, in addition to diffuse subcutaneous emphysema, a large pneumomediastinum, and a left pneumothorax. The surgery consisted of a left cervicotomy, a pharyngeal incision, and retrieval of the trapped parts. The patient was sent to the intensive care unit (ICU) where she could be weaned and extubated 1 week later.

## Case presentation

### Introduction

Ensuring proper nutritional status in patients with chronic obstructive pulmonary disease (COPD) prior to lung transplantation is crucial for enhancing postoperative outcomes (1). Individuals who are unable to meet their caloric needs through oral intake may be considered for enteral nutrition as a viable option (2).

Percutaneous endoscopic gastrostomy (PEG) has become a commonly used method for enteral feeding. Despite its reputation as a safe and minimally invasive technique, complications can occur at any stage of the procedure, from insertion to removal (3) (4).

A 60-year-old female patient was admitted to our hospital for removal of her percutaneous endoscopic gastrostomy (PEG) feeding tube (MIC* PEG kit with an ENFit^®^ connector of 20 Fr; pull method; AVANOS), which was inserted 3 months prior to improve her nutritional status. She has advanced-stage COPD, and she was on triple inhaler therapy, home oxygen, and nocturnal non-invasive ventilation. She was on the waiting list for a lung transplant. She had no other relevant medical history. The PEG removal was planned to be done using an endoscopic approach according to our local hospital policy.

On admission, the patient was stable, saturating well on the nasal cannula. She was comfortable and had no signs of respiratory distress. An upper gastroscopy was performed without general anesthesia. During the procedure, which had been well tolerated so far, the stomach and the esophagus were inspected and found intact. The external part of the PEG tube was cut at the skin level, then a snare was introduced to grasp the internal bumper and pull it up using the gastroscope; however, once it reached the proximal end of the esophagus, the tube rotated transversally and became blocked at the level of the pharyngeal wall. The endoscopist was unable to remove it despite an increased effort. The patient started to experience respiratory distress and profound hypoxemia. A subcutaneous emphysema appeared, indicating a perforation had occurred. The patient was intubated rapidly but with difficulty owing to extrinsic compression of the airway by the PEG tube.

After stabilization, another endoscopic removal was attempted using a snare and forceps but failed. During this second attempt, we could visualize a 2.5 cm tear in the left pharyngeal wall.

**Figure 1:**
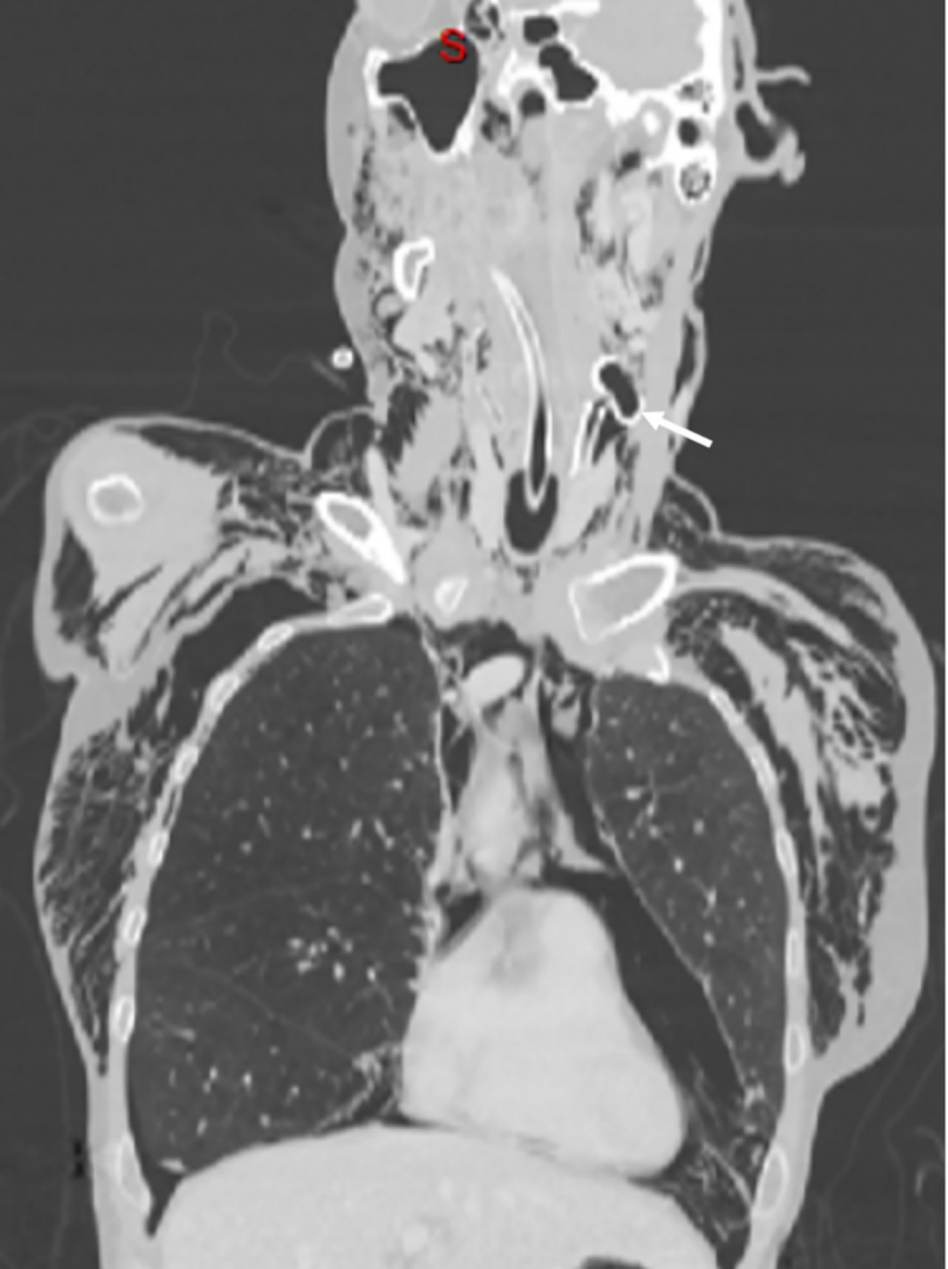
Coronal CT cut image showing the trapped PEG tube in the left cervical wall (arrow), cervical and thoracic subcutaneous emphysema, pneumomediastinum, and left pneumothorax. PEG, percutaneous endoscopic gastrostomy.

A CT scan was performed. The scan showed the piece blocked across the left cervical wall in proximity to the carotid triangle, a large non-tension pneumomediastinum dissecting the cervical tissues, and a mild left pneumothorax (**Figure 1**).

**Figure 2:**
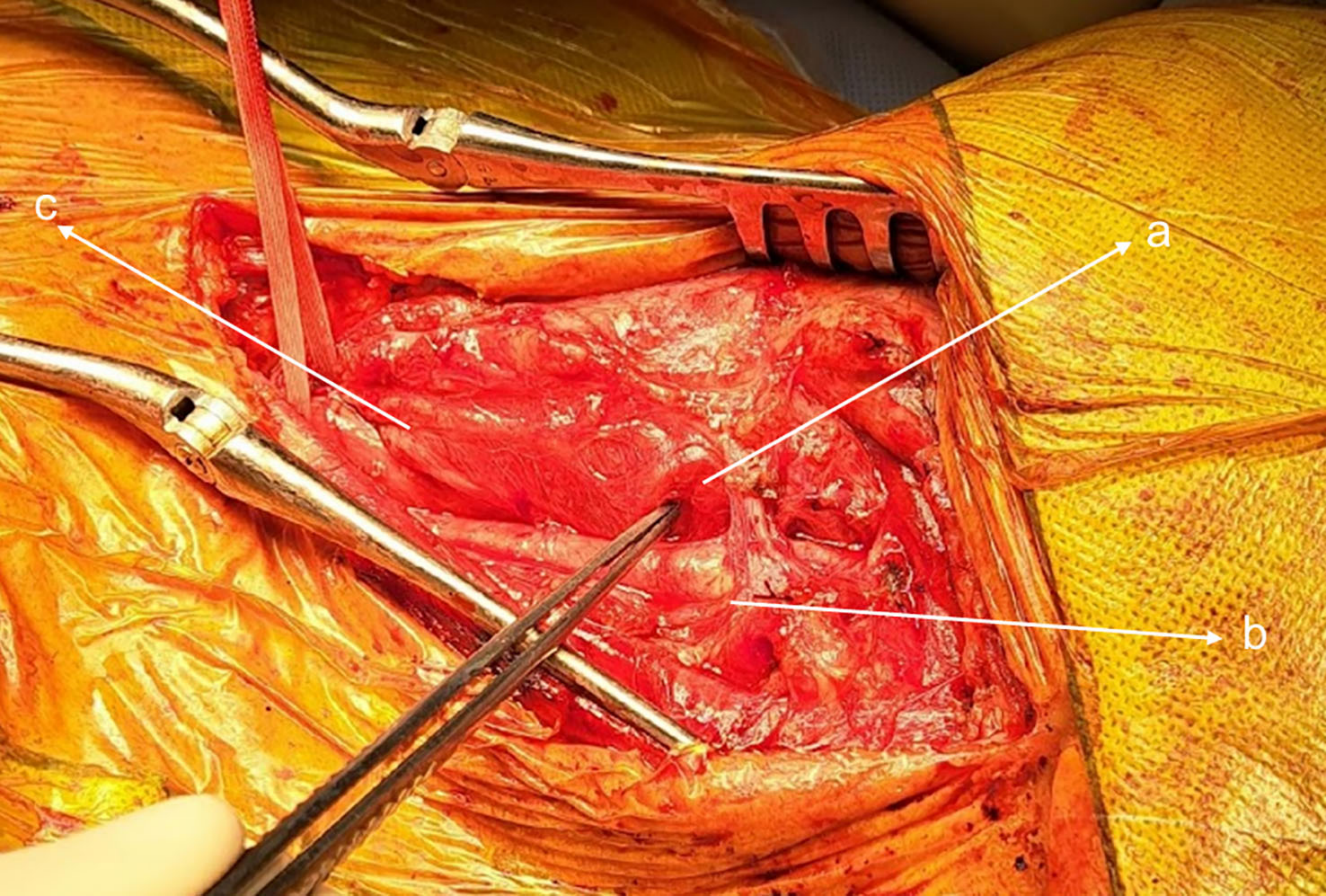
Left cervical incision: (a) blocked piece, (b) carotid artery, and (c) esophagus.

**Figure 3:**
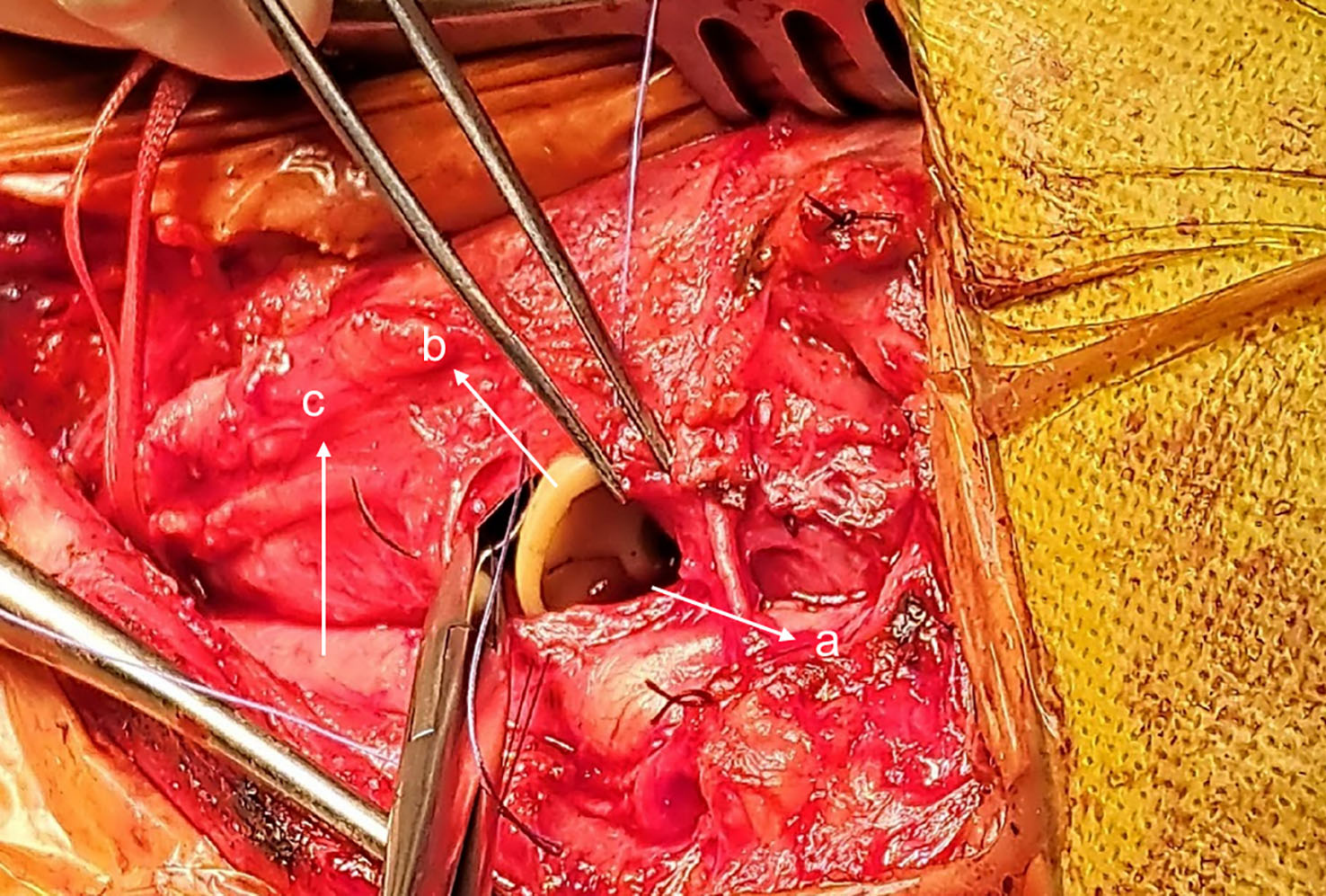
(a) Pharyngeal incision, (b) Trapped PEG and (c) Carotid artery.

**Figure 4:**
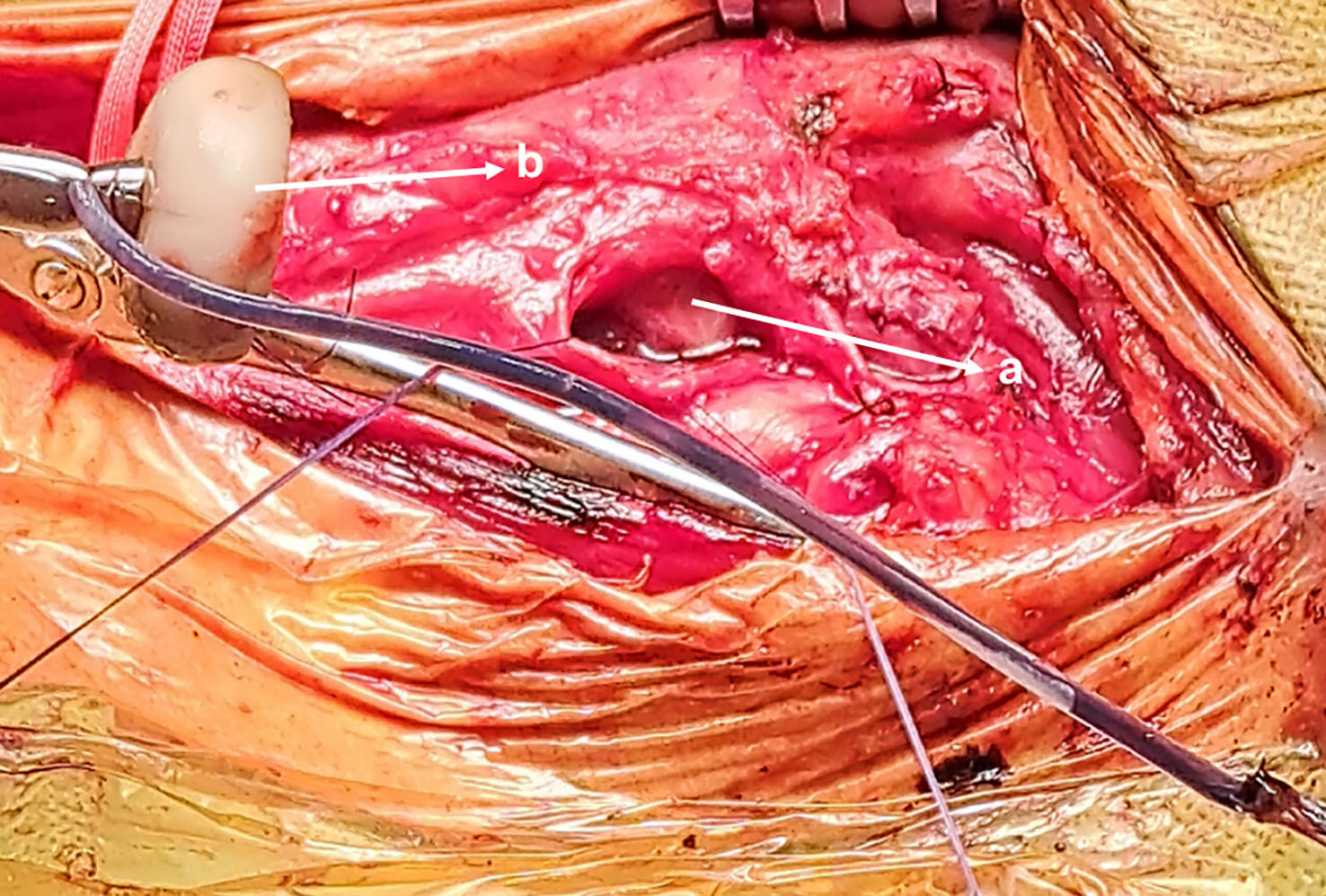
Removed PEG tube (a), Pharyngeal incision (b).

The patient was sent for surgical extraction. A left cervical incision was made along the sternocleidomastoid muscle. Exploration showed significant tissue inflammation and a difficult dissection owing to subcutaneous emphysema. The esophagus and carotid were successfully isolated, and the bumper was located in the pharynx above the Killian’s triangle (**Figures 2** and **3**). A longitudinal pharyngeal incision of 7 cm was required to remove the specimen, as mobilization was not possible (**Figure 4**). The wound was then closed, and a Penrose drain was placed. No chest tube was inserted as her respiratory condition stabilized on intubation and the tear was closed surgically.

The patient was sent back to the intensive care unit (ICU) where she was kept sedated, intubated, nil per os (NPO) and parenterally fed. Empirical antibiotics covering the oropharyngeal flora (metronidazole, aztreonam, and vancomycin) were started, taking into consideration the patient’s allergy to penicillin. Her ICU stay was uneventful. A control CT scan on day 6 revealed a healed wound without residual fistula and a resolution of the pneumothorax and the pneumomediastinum. The patient was successfully extubated 1 week later, and her home non-invasive positive pressure ventilation could be resumed without complication. She was transferred to the regular ward on day 12.

### Discussion

There are several approaches to removing a PEG, including endoscopic, cut and push, or simple traction. Each method has its potential advantages and adverse events, and the choice of the method depends on several factors such as local institutional preference, expertise, the type of the tube, and the insertion procedure (3) (4).

In the adult population, the endoscopic approach has become less popular because of its cost and the need for an endoscopist, whereas the other techniques are considered simpler and less invasive. However, endoscopic retrieval remains the gold standard for the pediatric population (4). Our local hospital policy was endoscopic removal.

Removing the intragastric device by endoscopic pulling can result in lacerations and perforations along the way. A few cases have been reported in the literature, with various clinical presentations ranging from asymptomatic subcutaneous emphysema (5) to severe respiratory distress (6). The authors identified several risk factors associated with these complications, such as a sharp tube edge, a rigid tube that was left in place for a long duration and became colonized with fungi (7), and the presence of osteophytes (5). Among the reported cases, only one required surgical extraction to remove the trapped piece (5).

Our patient had no known risk factors that might predispose her to such complications. She had not undergone any prior surgery or irradiation at the level of the neck. In addition, she did not have any kind of vertebral anomaly or deformity. Furthermore, the esophagus was inspected at the beginning of the procedure and was intact. In addition to this, the tube was not left in place for a prolonged period and did not appear to be infected or rigid on retrieval.

We believe that, in the case of our patient, the probable explanation is that on grasping the PEG, the snare held the tube near the unfolded bumper, exposing its sharp end. This caused the PEG to pivot, lacerating the pharyngeal mucosa, thus becoming embedded in the cervical soft tissue. Another attempt to remove it likely resulted in further damage.

Following this complication, our hospital policy concerning PEG tube removal was modified. The external traction and the cut and push approaches were favored over the endoscopic. The choice has to take into consideration the type of the tube, and the presence of any contraindications. In a case in which the endoscopic technique was used as a last resort, the decision was made to grasp the tube 3 cm away from the bumper to minimize the risk of rotation and the risk of blockage.

### Conclusion

Endoscopic removal of the PEG tube can result in lacerations along the way that may lead to serious thoracic complications. When this method is used, precautions should be taken to minimize these risks, such as the use of a tube cover to protect the mucosa and holding the tube at least 3 cm away from the bumper.

It is important to prioritize less invasive removal methods, such as cut and push or simple extraction, particularly in cases involving a rigid tube, difficult anatomy, or frail respiratory status, to minimize the risk of complications.

## Data availability statement

The original contributions presented in the study are included in the article/supplementary material. Further inquiries can be directed to the corresponding author.

## Ethics statement

The studies involving human participants were reviewed and approved by Groupe Hospitalier de la Région de Mulhouse et Sud Alsace, unité de recherche clinique. The patients/participants provided their written informed consent to participate in this study. Written informed consent was obtained from the individual(s) for the publication of any potentially identifiable images or data included in this article.

## Author contributions

The authors confirm their contribution to all parts of the papers including: the literature search, data collection, and manuscript preparation and manuscript review. The authors agreed to be accountable for all aspects of the work in ensuring that questions related to the accuracy or integrity of any part of the work are appropriately investigated and resolved. All authors contributed to the article and approved the submitted version.
